# Supplementary material for: Factors Influencing Virtual Art Therapy in Patients with Stroke
Source: Brain Sci. 2025 Jul 9;15(7):736. doi: 10.3390/brainsci15070736 (PMC12293237; doi:10.3390/brainsci15070736)
Supplement: Supplementary file 1 [file brainsci-15-00736-s001.zip › brainsci-3698297-supplementary.pdf]

List of stimuli

1. “Annunciation” by Leonardo Da Vinci
2. “Dance” by Henry Matisse
3. “The Creation of Adam” by Michelangelo Buonarroti
4. “The Calling of Saint Matthew” by Caravaggio
5. “The Luncheon of the Boating Party” by Auguste Renoir
6. “The Birth of Venus” by Sandro Botticelli
7. “The Great Wave” of Kanagawa
8. “The Night Café” by Vincent Van Gogh
9. “Three Musicians” by Pablo Picasso
10. “Bedroom in Arles” by Vincent Van Gogh
11. “The Kiss” by Gustav Klimt
12. “The Sacrament of the Last Supper” by Salvador Dalì
13. “The Studio Boat” by Claude Monet
14. “Flagellation of Christ” by Piero della Francesca
15. “A Sunday Afternoon on the Island of La Grande Jatte” by Georges Seurat
16. “The Night Watch” by Rembrandt
17. “The Starry Night” by Vincent Van Gogh
18. “The Parc Monceau” by Claude Monet
19. “The Water-Lily Pond” by Claude Monet
20. Self-Portrait by Freeda
21. “The Daydream” by Auguste Renoir
22. “The Son of Man” by René Magritte
23. “Sunflowers” by Vincent Van Gogh
24. “The Scream” by Edvard Munch
25. “Café Terrace at Night” by Vincent Van Gogh
26. “Vertumnus” by Giuseppe Arcimboldo
27. “The Kiss” by Francesco Hayez
28. “Mona Lisa” by Leonardo Da Vinci
29. “Lady with an Ermine” by Leonardo Da Vinci
30. “Girl with a Pearl Earring” by Johannes Vermeer
